# Supplementary material for: Microfluidics combined with electron microscopy for rapid and high-throughput mapping of antibody–viral glycoprotein complexes
Source: Nat Biomed Eng. 2025 Jun 3;9(11):1938–51. doi: 10.1038/s41551-025-01411-x (PMC12404239; doi:10.1038/s41551-025-01411-x)
Supplement: Supplementary file 1 — Supplementary Figs. 1–14 and Tables 1 and 2. [file 41551_2025_1411_MOESM1_ESM.pdf]

nature biomedical engineering

Supplementary information https://doi.org/10.1038/s41551-025-01411-x

Microfluidics combined with electron microscopy for rapid and high-throughput mapping of antibody–viral glycoprotein complexes

In the format provided by the authors and unedited

Supplementary Figures

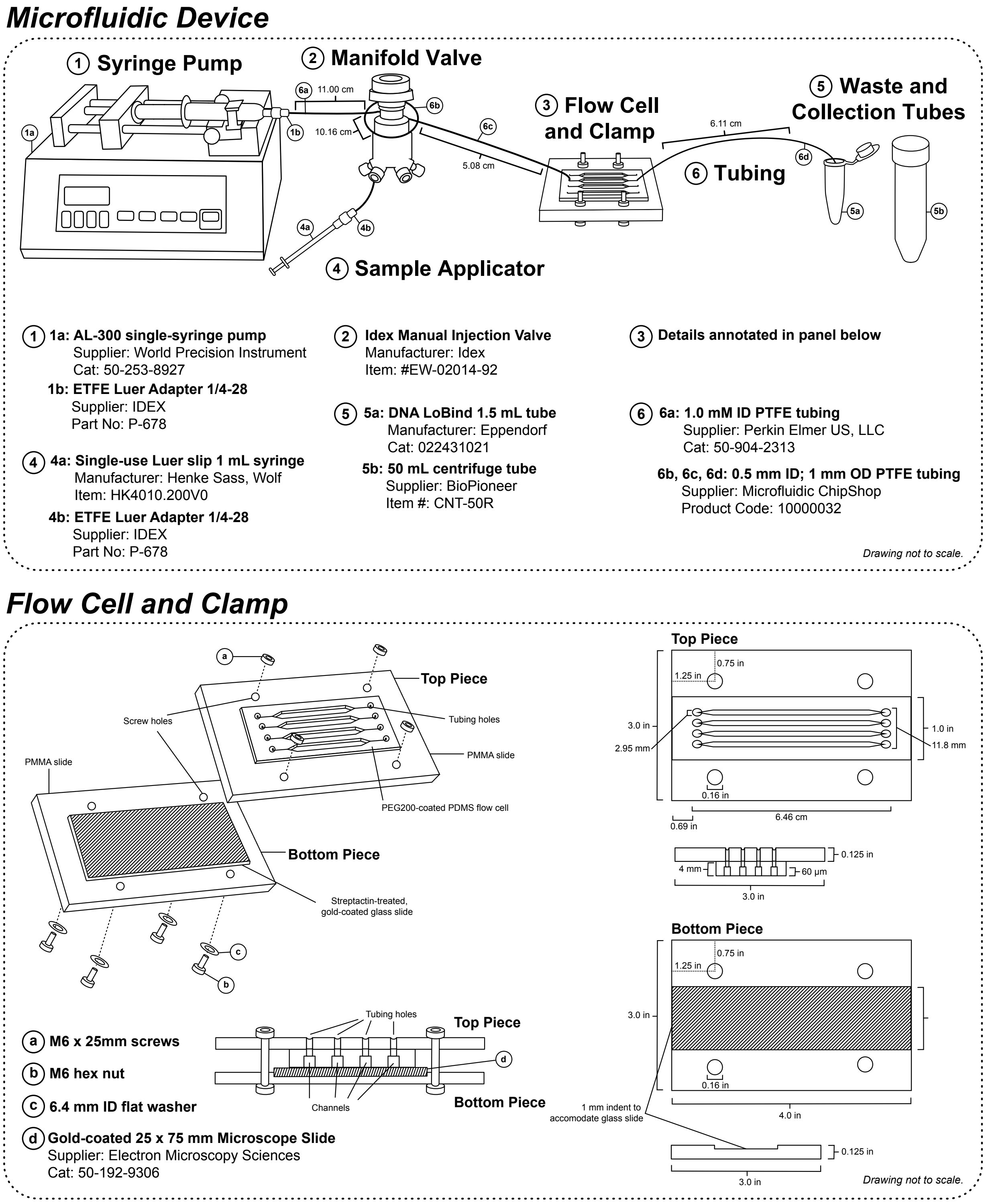

Figure S1. Design of mEM system. Technical drawings of microfluidic device including flow cell and clamp. Dimensions of each component and the parts used are listed.

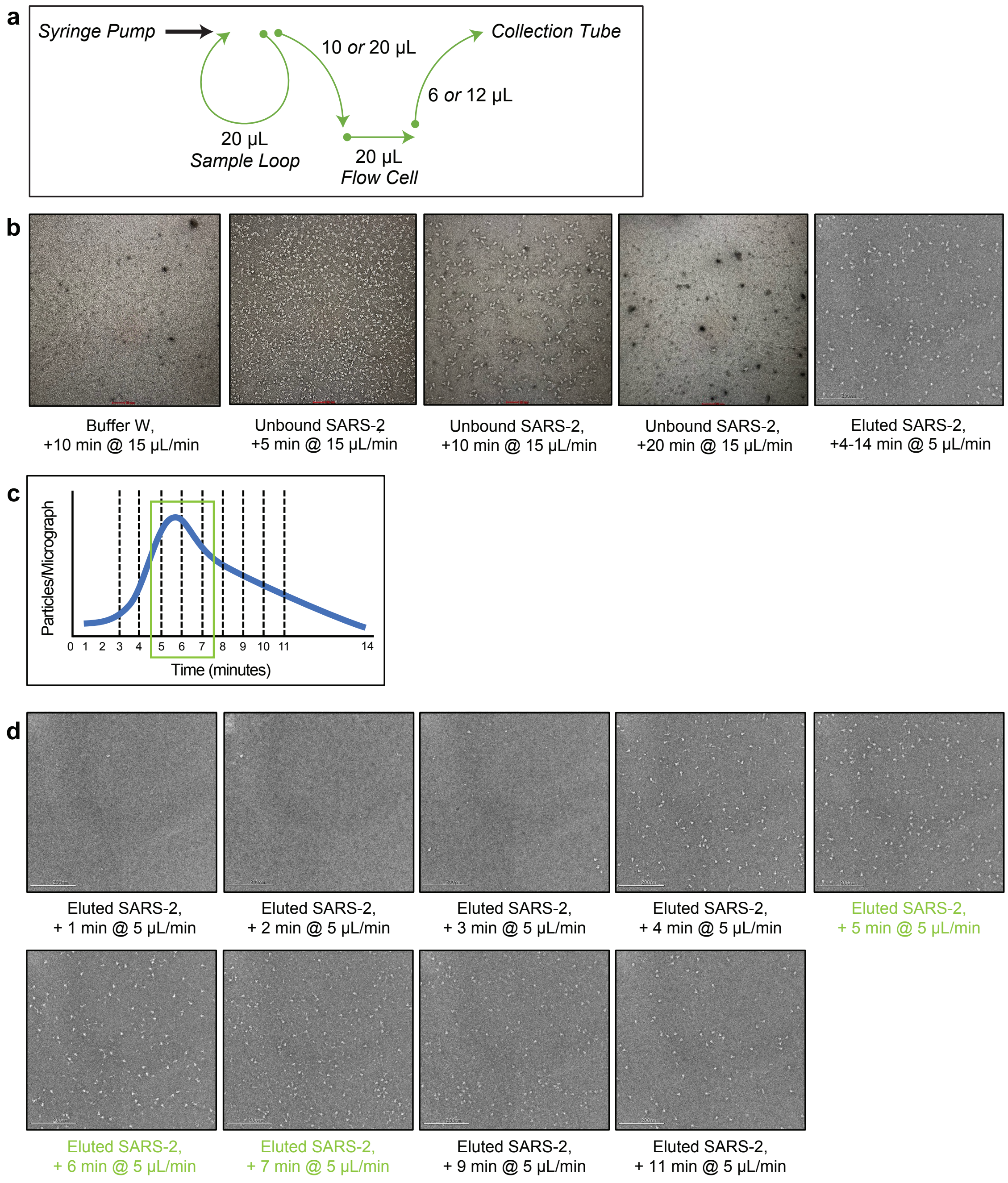

Figure S2. mEM system overview and elution timing. a, Schematic of sample movement through mEM microfluidic system and including tubing distances between each of the following components: syringe pump, sample loop, valve—flow cell connector, PDMS flow cell and exit tube. **b,** Timelapse imaging using ns-EM of SARS-2 Spike movement through mEM system, including before glycoprotein injection, following immobilization and glycoprotein elution. **c,** Cartoon graphic depicting collection of glycoprotein elution fractions from system exit between 0

and 14 minutes. Time in minutes is represented on the x-axis and particles per micrograph is shown on the y-axis. The illustrated elution profile is colored blue with the timing representing the highest proportion of particles boxed in green. **d,** Timelapse imaging of SARS-2 Spike elution from mEM system using ns-EM. Fractions were assessed every minute from 0-7 minutes followed by 2-minute increments until 11 minutes. System flow was set to 5 µL/min for collection.

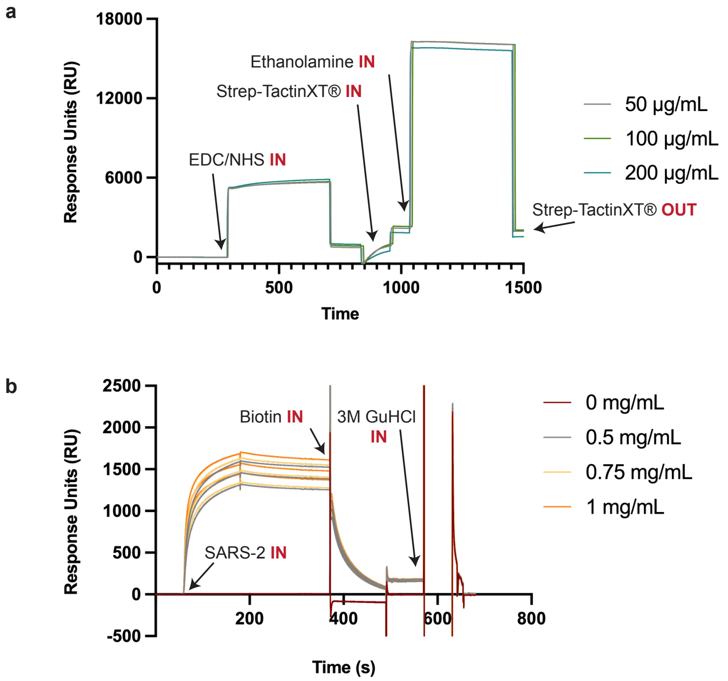

Figure S3. Validation of MHDA surface using SPR. a, Strep-Tactin®XT functionalized to 2.5 mM MHDA gold surface using amine coupling. Three concentrations of Strep-Tactin®XT were used, including 50, 100 and 200 µg/mL. Functionalization consisted of an injection of 50 µL of EDC/NHS followed by Strep-Tactin®XT and ethanolamine. **b,** SARS-CoV-2 Spike glycoprotein binding to the 100 µg/mL Strep-Tactin®XT surface was performed in triplicate. Different concentrations of Spike (0-1 mg/mL) were injected followed by an elution with Biotin. The surface was then regenerated (3M GuHCl) in between each injection. 10mM PBS pH 7.4 was used as a flow buffer at a flow rate of 15 µL/min.

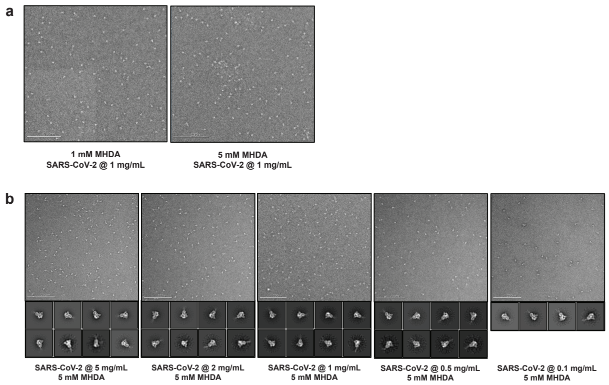

Figure S4. Optimization of glycoprotein immobilization using mEM. a, Sample ns-EM micrographs of SARS-CoV-2 Spike glycoprotein elution from gold surfaces prepared at two different MHDA concentrations: 1 mM MHDA (left panel) and 5 mM MHDA (right panel). Spikes appear as white particles on the dark background. In both conditions the glycoprotein was injected at 1 mg/mL and eluted at 5 µL/min with the elution placed immediately on an EM grid. **b,** Sample micrograph and 2D class averages of SARS-CoV-2 Spike glycoproteins eluted from mEM at different injection concentrations, including 5 mg/mL, 1 mg/mL, 0,5 mg/mL and 0.1 mg/mL. In all cases a concentration of 5 mM MHDA was used for biofunctionalization of gold surfaces.

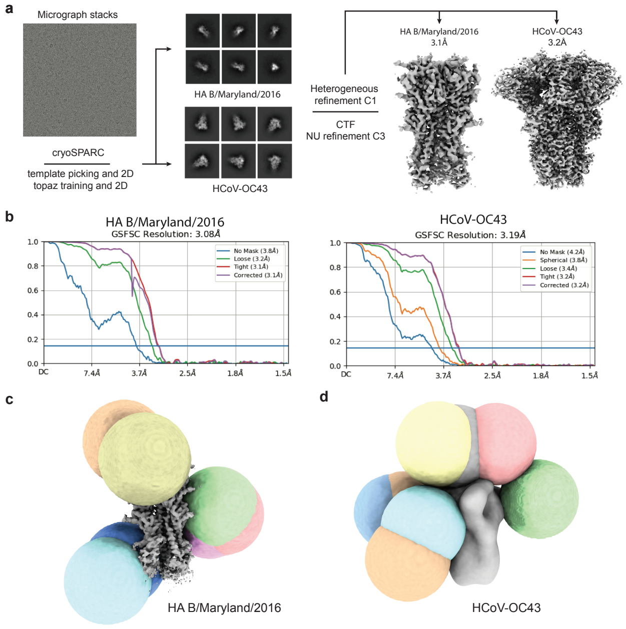

Figure S5. Cryo-EM processing workflow. a, Schematic representation of the cryo-EM data processing workflow showing the individual cryo-EM maps of HA B/Maryland/2016 and HCoV OC43. **b,** Fourier shell correlation (FSC) plots corresponding to the reconstructions shown in panel A. **c,** Masks used for focus classification of the two cryo-EMPEM datasets: HA complexed with Donor 2323 Day 7 purified Fabs and HCoV OC43 complexed with Donor 2327 Day 2 sera.

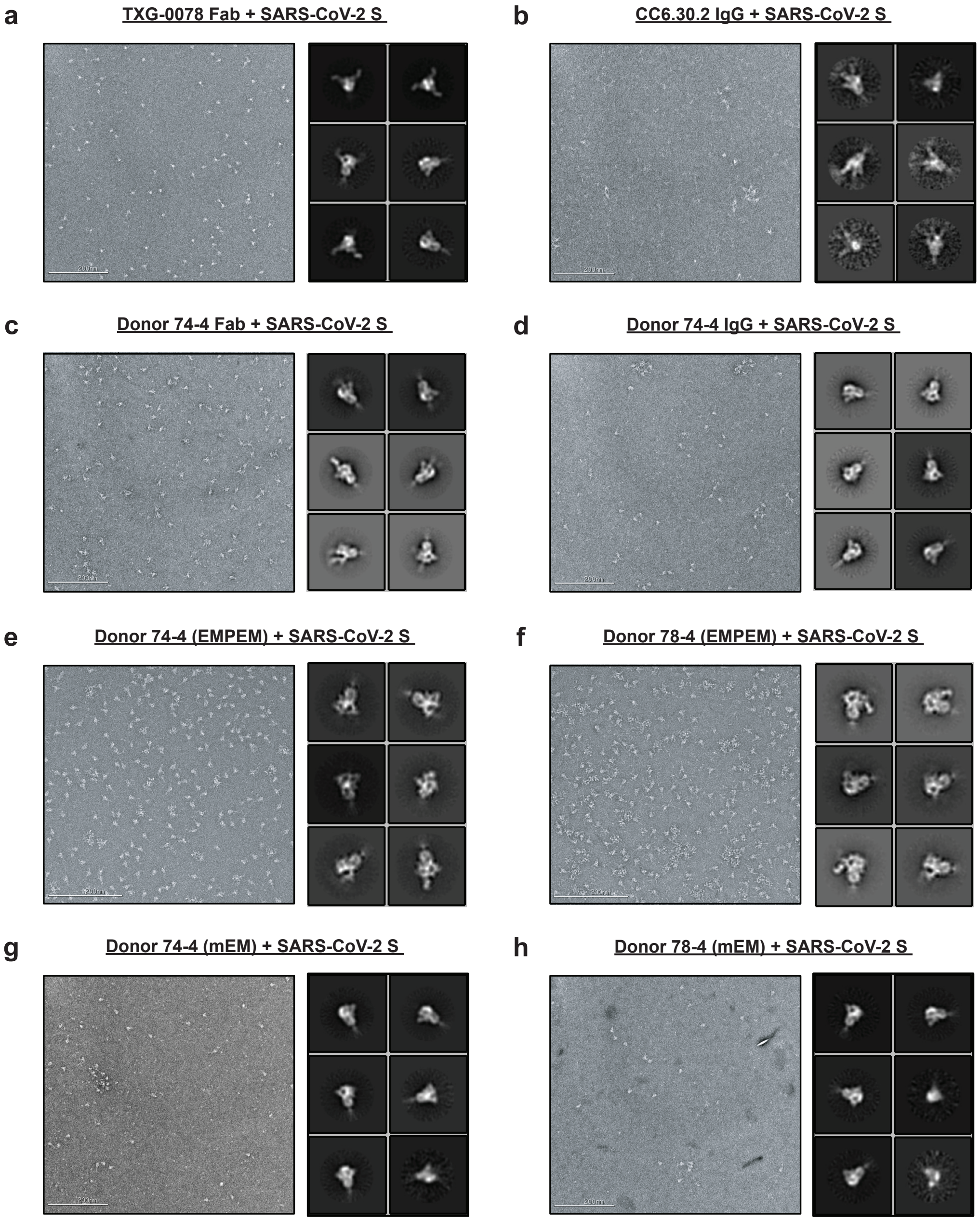

Figure S6. Extended ns-EM analysis for SARS-CoV-2 Spike-antibody complexes. Representative micrographs and 2D class averages from the ns-EM datasets used for the generation of composite figures presented in Figure 3.

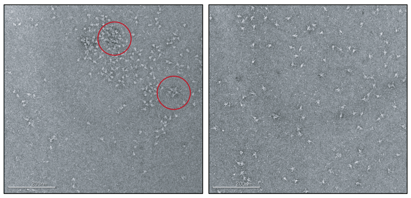

Figure S7. Polyclonal IgG induces aggregate formation compared to Fab. Representative micrographs of purified polyclonal IgG (left panel) and polyclonal Fab (right panel) from Donor 74 complexed with SARS-CoV-2 Spike glycoprotein using mEM. Aggregates formed when polyclonal IgG is used for complexing are circled in red.

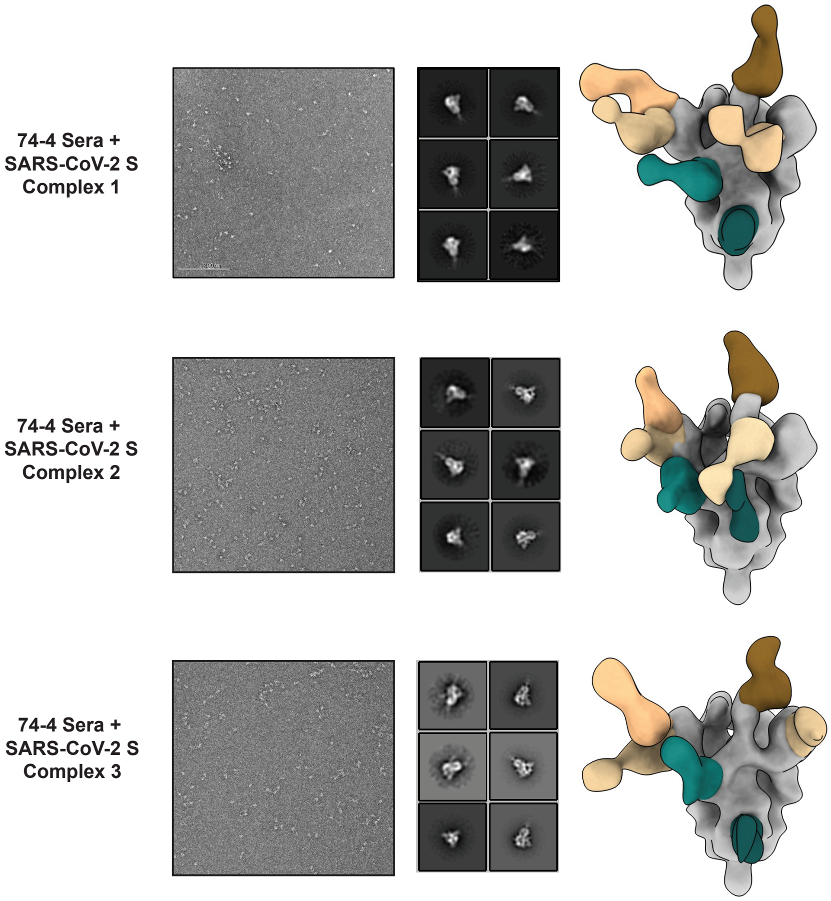

Figure S8. ns-EM analysis of Donor 74-4 in complex with SARS-CoV-2 S. Sera from Donor 74-4 was complexed in triplicate using mEM and final 3D reconstructions of polyclonal antibodies bound to S were compared. Representative micrographs, 2D class averages and 3D composite maps for the three complexes are shown.

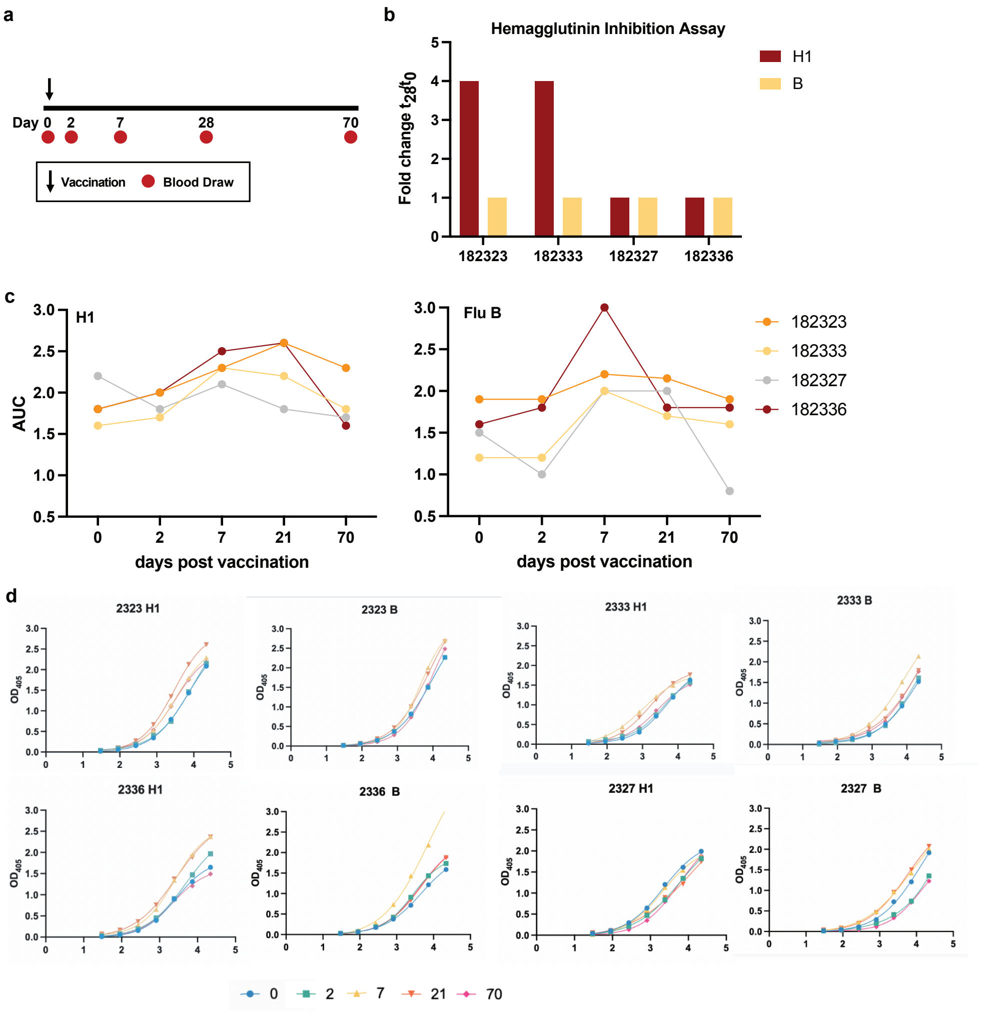

Figure S9. Detection of polyclonal antibody response against hemagglutinin after vaccination. a, Vaccination schedule. Red arrows represent the weeks that blood was drawn and black arrows when individuals were immunized with the flu vaccine Fluzone 2018-2019. **b,** Fold change detected by Hemagglutinin Inhibition assays between day 28 after vaccination and day 0 pre-vaccination for H1 and B/Maryland/2016. **c,** IgG midpoint titers over time determined via ELISA**. d,** Anti-HA binding antibody titers (EC_{50}) determined by ELISA for sera samples at different timepoints. X axis represents sera dilutions in logarithmic scale. Sera was tested against recombinantly expressed hemagglutinin from A/Michigan/2015 and B/Maryland/2016 strains.

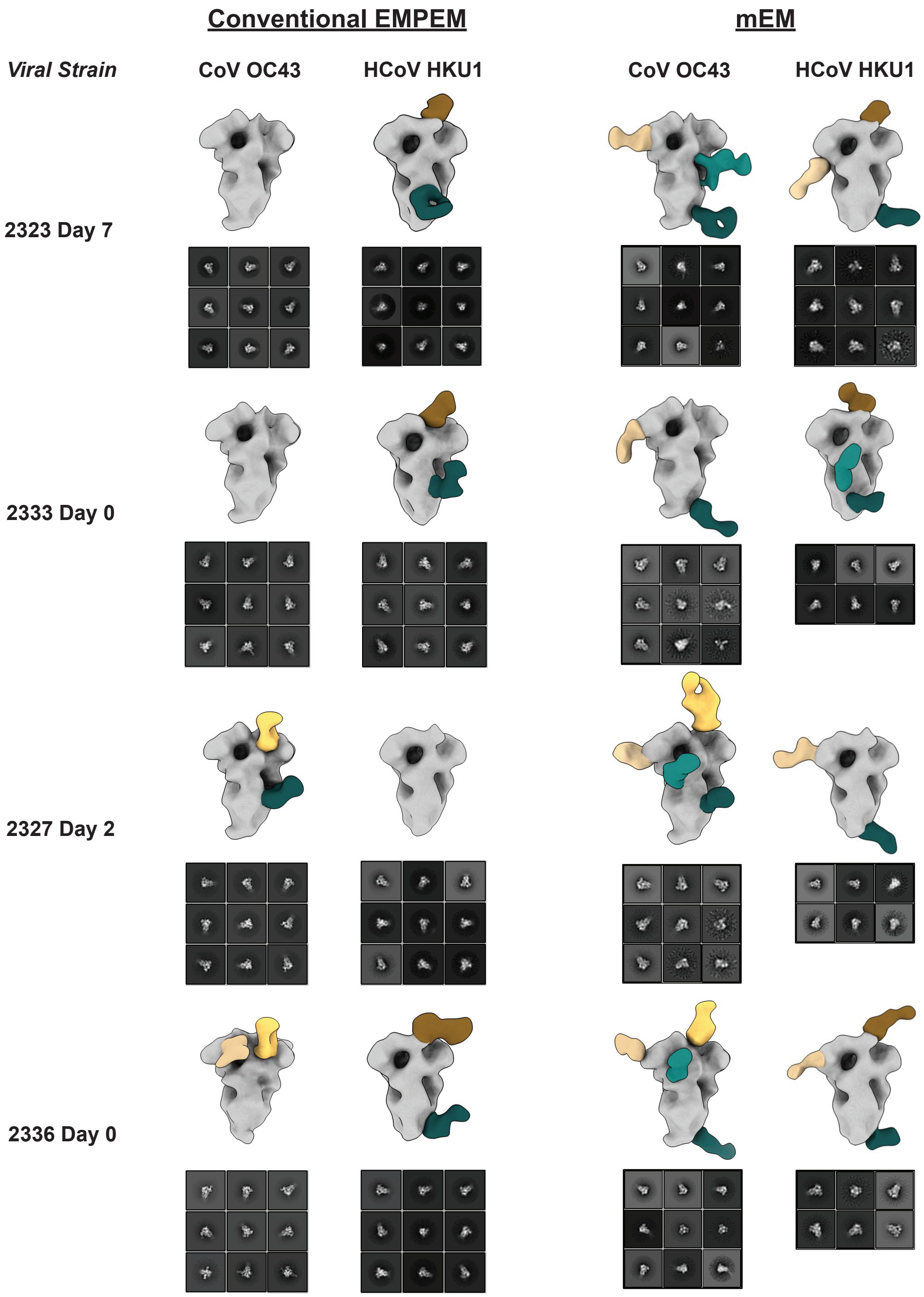

Figure S10. Extended ns-EM analysis for CoV memory responses. Representative 2D class averages and 3D composite maps used for the generation of the CoV Spike dot plot figures presented in Figure 4.

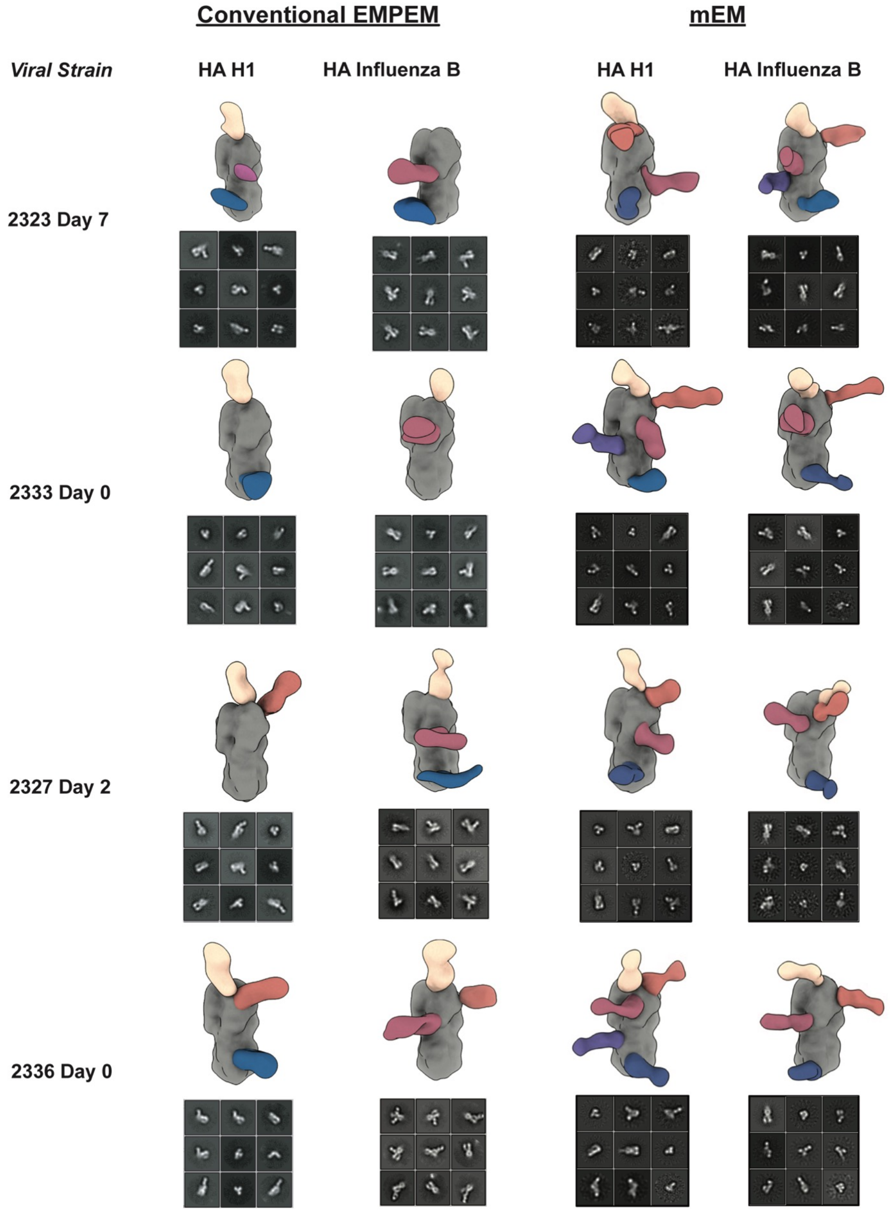

Figure S11. Extended ns-EM analysis for vaccine-elicited responses against Influenza HA. Representative 2D class averages and 3D composite maps used for the generation of the Influenza HA dot plot figures presented in Figure 4.

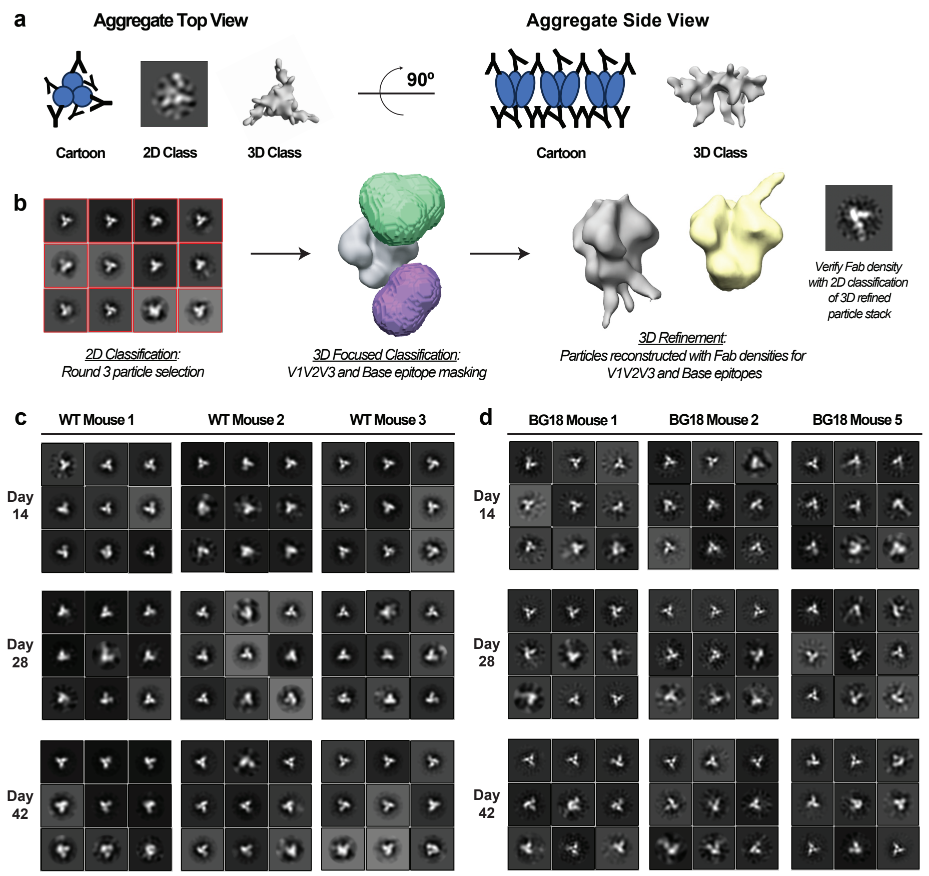

Figure S12. Extended ns-EM analysis by mEM of vaccine-elicited responses against HIV Env N332-GT5. a, Cartoon depiction of HIV Env-antibody aggregate formation when using a higher plasma dilution. Representative 2D class average and 3D classification (top and side views) of the aggregate particles are shown in gray. **b,** Schematic representation of the ns-EM data processing workflow showing particle selection after round 3 of 2D classification, epitope masking using 3D focused classification (green = V1V2V3 epitope, purple = base epitope) and final 3D refinement of the selected classes with Fab densities. Final verification is done using 2D classification of the 3D refined maps to ensure antibody density is present. **c-d,** Representative 2D class averages used for the generation of composite figures presented in Figure 5.

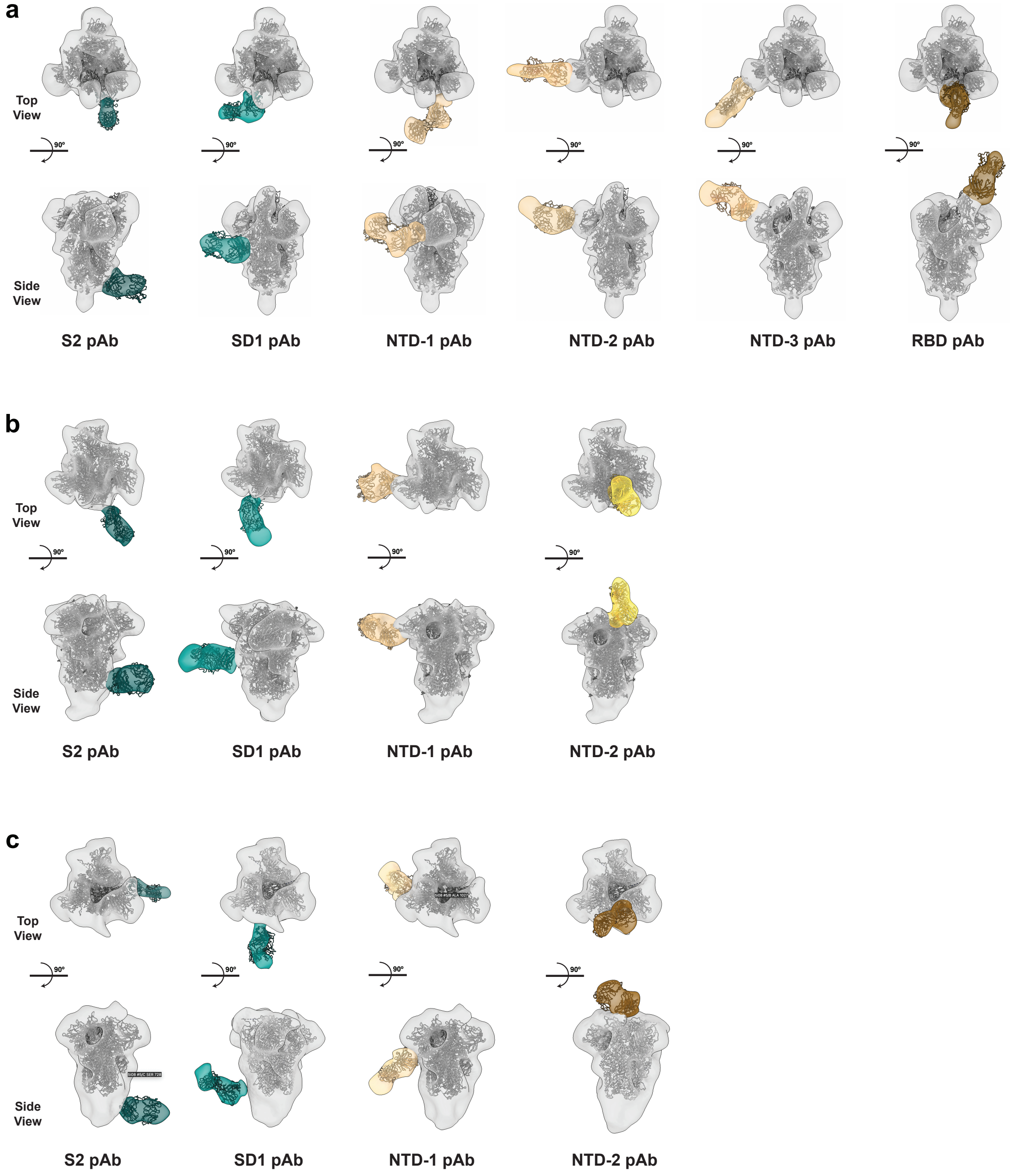

Figure S13. Rigid-body fits of SARS-CoV-2, OC43 and HKU1 Coronavirus Spike glycoproteins with corresponding polyclonal Fab (pFab) densities. a, Models of SARS-CoV-2 S (PDB 6VYB), **b**, OC43 S (PDB 6OHW), and **c**, HKU1 S (PDB 5I08) were docked into the low pass filtered trimer densities and are colored gray. For all polyclonal epitopes determined for each S glycoprotein, a human polyclonal Fab model with a polyalanine backbone was docked into the

Fab densities to determine relative fit. Antibodies representative of the detected epitopes are shown from donors 74-4 (**a**), 2327 (**b**), 2323 (**c**, S2 and NTD) and 2333 (**c**, SD2 and RBD) with the Fab densities and models colored the same as in Figure 4. Top and side views for all complexes are shown.

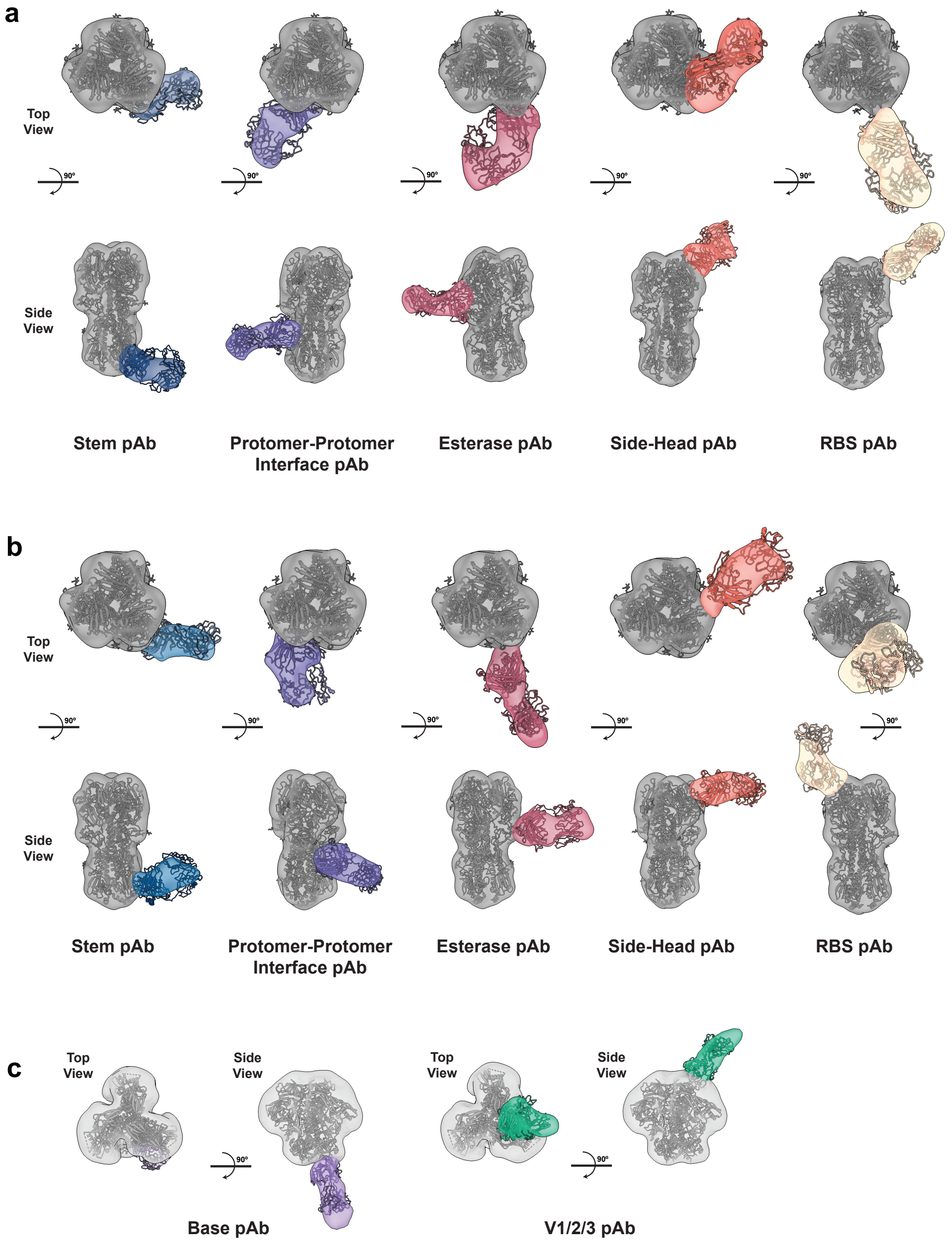

Figure S14. Rigid-body fits of influenza HA H1/Michigan/2015 and B/Maryland/2016, and HIV Env N332-GT5 glycoproteins with corresponding polyclonal Fab (pFab) densities. a-b, Models of HA H1 and HA Influenza B (PDB 7KNA), HA Influenza B (PDB 7KNA), and **c**, HIV Env N332-GT5 (PDB 8T4K) were docked into the low pass filtered trimer densities and are colored gray. For all polyclonal epitopes determined for each HA and HIV Env glycoprotein, a human polyclonal Fab model with a polyalanine backbone was docked into the Fab densities to determine

relative fit. Antibodies representative of the detected epitopes are shown from donors 2336 (**a**), 2323 (**b**), Mouse Group 1 #3 Week 42 (**c**) with the Fab densities and models colored the same as in Figure 4 and 5. Top and side views for all complexes are shown.

Table S1. Cryo-EM data collection, refinement and validation statistics

	HCoV OC43 S protein (EMDB-44658) (PDB 9BLK)	HA B/Maryland/2016 (EMDB-44894) (PDB 9BTO)	SARS-CoV-2 spike with all RBD down (EMD-44679)	SARS-CoV-2 spike glycoprotein with 1 RBD up (EMDB-44680)	2327 day 7 sera + HCoV OC43 S protein (EMDB-44882)	2323 day 7 Fab + HA B/Maryland/2016 (EMDB- 44883)
**Data collection and processing**						
Microscope	TFS Glacios	TFS Glacios	FEI Talos Arctica	FEI Talos Arctica	TFS Glacios	TFS Glacios
Magnification	190,000x	190,000x	36,000	36,000	190,000	190,000
Voltage (kV)	200	200	200	200	200	200
Electron exposure (e–/Å^2)	50	50	50	50	50	50
Defocus range (μm)	-0.8 to -1.8	-0.8 to -1.8	-1.0 to -2.0	-1.0 to -2.0	-0.8 to -1.8	-0.8 to -1.8
Detector	TFS Falcon IV	TFS Falcon IV	Gatan K2 Summit	Gatan K2 Summit	TFS Falcon IVi	TFS Falcon IV
Recording mode	Counting	Counting	Counting	Counting	Counting	Counting
Pixel size (Å)	0.725	0.725	1.15	1.15	0.718	0.725
Symmetry imposed	C3	C3	C3	C1	C1	C1
Micrographs (no.)	815	815	2,983	2,983	7,344	8,518
Initial particle images (no.)	120,002	120,002	40,468	40,468	106,745	204,392
Final particle images (no.)	18,599	16,096	20,588	9,755	449	549
Map resolution (Å)	3.3	3.1	4.6	6.9	24.9	26.0
FSC threshold	0.143	0.143	0.143	0.143	0.143	0.143
Map sharpening *B* factor (Å^2)	-51.3	-62.6	-178.4	-476.3	N/A	N/A
Map pixel size (Å)	0.725	0.725	1.15	1.15	0.933	0.725
Map resolution range (Å)	2.8-4.2	2.4-4.5	4.1-5.6	6.2-8.0	N/A	N/A
**Refinement**						
Initial model used (PDB code)	6X9R		N/A	N/A	N/A	N/A
Model resolution (Å)	3.3	3.2	N/A	N/A	N/A	N/A
FSC threshold	0.5	0.5	N/A	N/A	N/A	N/A
Model resolution range (Å)	2.8-4.2	2.4-4.5	N/A	N/A	N/A	N/A
EMRinger score	3.23	4.17	N/A	N/A	N/A	N/A
Model composition						
Non-hydrogen atoms	28,035	11,586	N/A	N/A	N/A	N/A
Protein residues	3,519	1,437	N/A	N/A	N/A	N/A
Ligands	45	51	N/A	N/A	N/A	N/A
Mean *B* factors (Å^2)						
Protein	28.84	112.55	N/A	N/A	N/A	N/A
Ligand	45.60	139.02	N/A	N/A	N/A	N/A
R.m.s. deviations						
Bond lengths (Å)	0.021	0.023	N/A	N/A	N/A	N/A
Bond angles (°)	1.777	2.012	N/A	N/A	N/A	N/A
Validation						
MolProbity score	0.85	0.61	N/A	N/A	N/A	N/A
Clashscore	0.67	0.30	N/A	N/A	N/A	N/A
Poor rotamers (%)	0.10	0.00	N/A	N/A	N/A	N/A
Ramachandran plot						
Favored (%)	97.33	98.11	N/A	N/A	N/A	N/A
Allowed (%)	2.67	1.89	N/A	N/A	N/A	N/A
Disallowed (%)	0.00	0.00	N/A	N/A	N/A	N/A

Table S2. Ns-EM Electron Microscopy Data Bank deposition information. Maps are accessible at emdataresouce.org using the listed codes. Additional maps can be found on the "Download" tab of each entry.

EMDB Code	Method	Glycoprotein	Donor ID	Map (C1 symmetry)	Polyclonal Antibody Epitope	Contour
**EMD-44655**	Conventional EMPEM	HA H1	2323	Main Map + Half Maps 1 & 2	RBS	0.03
			2327	Additional Map	Side Head	0.03
			2323	Additional Map	Esterase	0.03
			2323	Additional Map	Stem	0.03
**EMD-44656**	Conventional EMPEM	HA Influenza B	2336	Main Map + Half Maps 1 & 2	RBS	0.03
			2336	Additional Map	Side Head	0.03
			2336	Additional Map	Esterase	0.03
			2327	Additional Map	Stem	0.03
**EMD-44657**	Conventional EMPEM	CoV OC43 S	2336	Main Map + Half Maps 1 & 2	Interface	0.03
			2336	Additional Map	NTD	0.03
			2327	Additional Map	S2	0.03
**EMD-44659**	Conventional EMPEM	CoV HKU1 S	2333	Main Map + Half Maps 1 & 2	CTD	0.03
			2333	Additional Map	SD2	0.03
			2323	Additional Map	S2	0.03
**EMD-44660**	Conventional EMPEM	SARS-CoV-2 S	74	Main Map + Half Maps 1 & 2	RBD	0.03
			74	Additional Map	NTD-1, NTD-3	0.03
			74	Additional Map	NTD-2	0.03
			74	Additional Map	S2	0.03
**EMD-44661**	mEM	HA H1	2333	Main Map + Half Maps 1 & 2	RBS	0.03
			2333	Additional Map	Side Head	0.03
			2333	Additional Map	Esterase	0.03
			2333	Additional Map	Protomer-protomer interface	0.03
			2333	Additional Map	Stem	0.03
**EMD-44662**	mEM	HA Influenza B	2323	Main Map + Half Maps 1 & 2	RBS	0.03
			2323	Additional Map	Side Head	0.03
			2323	Additional Map	Esterase, Protomer-protomer interface	0.03
			2323	Additional Map	Stem	0.03
**EMD-44663**	mEM	CoV OC43 S	2327	Main Map + Half Maps 1 & 2	Interface	0.03
			2327	Additional Map	NTD	0.03
			2327	Additional Map	SD2	0.03
			2327	Additional Map	S2	0.03
**EMD-44664**	mEM	CoV HKU1 S	2323	Main Map + Half Maps 1 & 2	CTD	0.03
			2323	Additional Map	NTD, S2	0.03
			2333	Additional Map	SD2	0.03
**EMD-44665**	mEM	SARS-CoV-2 S	74	Main Map + Half Maps 1 & 2	RBD	0.01
			74	Additional Map	NTD-1	0.01
			74	Additional Map	NTD-2	0.01
			74	Additional Map	NTD-3, SD2, S2	0.01
**EMD-44669**	mEM	HIV Env N332-GT5	Grp 1, Mouse 3, Day 42	Main Map + Half Maps 1 & 2	V1/V3	0.03
			Grp 1, Mouse 3, Day 42	Additional Map	Base	0.03
**EMD-44670**	mEM	HIV Env N332-GT5	Grp 2, Mouse 5, Day 42	Main Map + Half Maps 1 & 2	V1/V3	0.03
			Grp 2, Mouse 5, Day 42	Additional Map	Base	0.03

EMDB Code	Method	Glycoprotein	Monoclonal Antibody	Map (C1symmetry)	Contour
**EMD-44667**	mEM	SARS-CoV-2 S	TXG-0078	Main Map + Half Maps 1 & 2	0.03
**EMD-44668**	MEM	SARS-CoV-2 S	CC6.30.2	Main Map + Half Maps 1 & 2	0.03
