# Supplementary material for: Microfluidics combined with electron microscopy for rapid and high-throughput mapping of antibody–viral glycoprotein complexes
Source: Nat Biomed Eng. 2025 Jun 3;9(11):1938–51. doi: 10.1038/s41551-025-01411-x (PMC12404239; doi:10.1038/s41551-025-01411-x)
Supplement: Supplementary file 2 — Reporting Summary [file 41551_2025_1411_MOESM2_ESM.pdf]

nature portfolio

Corresponding author(s): Andrew B Ward

Last updated by author(s): Mar 31, 2025

Reporting Summary

Nature Portfolio wishes to improve the reproducibility of the work that we publish. This form provides structure for consistency and transparency in reporting. For further information on Nature Portfolio policies, see our Editorial Policies and the Editorial Policy Checklist.

Statistics

For all statistical analyses, confirm that the following items are present in the figure legend, table legend, main text, or Methods section.

n/a	Confirmed	
☐	☒	The exact sample size (*n*) for each experimental group/condition, given as a discrete number and unit of measurement
☐	☒	A statement on whether measurements were taken from distinct samples or whether the same sample was measured repeatedly
☒	☐	The statistical test(s) used AND whether they are one- or two-sided<br>*Only common tests should be described solely by name; describe more complex techniques in the Methods section.*
☒	☐	A description of all covariates tested
☒	☐	A description of any assumptions or corrections, such as tests of normality and adjustment for multiple comparisons
☐	☒	A full description of the statistical parameters including central tendency (e.g. means) or other basic estimates (e.g. regression coefficient) AND variation (e.g. standard deviation) or associated estimates of uncertainty (e.g. confidence intervals)
☒	☐	For null hypothesis testing, the test statistic (e.g. *F*, *t*, *r*) with confidence intervals, effect sizes, degrees of freedom and *P* value noted<br>*Give P values as exact values whenever suitable.*
☒	☐	For Bayesian analysis, information on the choice of priors and Markov chain Monte Carlo settings
☒	☐	For hierarchical and complex designs, identification of the appropriate level for tests and full reporting of outcomes
☒	☐	Estimates of effect sizes (e.g. Cohen's *d*, Pearson's *r*), indicating how they were calculated

Our web collection on statistics for biologists contains articles on many of the points above.

Software and code

Policy information about availability of computer code

Data collection	Leginon (beta version), EPU (ThermoFisher)
Data analysis	Relion (v3.0 & v4.0), CryoSPARC (v2.15), Octet System Data Analysis (v9.0), Excel (v16.43), GraphPad Prism (v8.4.3), Appion (v1), UCSF Chimera (v1.13), MotionCor (v2), MolProbity (v4.2), EMRinger (version N/A), Localized Reconstruction (v1.2.0), GCTF (v1.06_sm_30_cu8.0_x86_64), Coot (v0.9-pre), BIAEvaluation (1.1.1)

For manuscripts utilizing custom algorithms or software that are central to the research but not yet described in published literature, software must be made available to editors and reviewers. We strongly encourage code deposition in a community repository (e.g. GitHub). See the Nature Portfolio guidelines for submitting code & software for further information.

Data

Policy information about availability of data

All manuscripts must include a data availability statement. This statement should provide the following information, where applicable:

- Accession codes, unique identifiers, or web links for publicly available datasets
- A description of any restrictions on data availability
- For clinical datasets or third party data, please ensure that the statement adheres to our policy

3D maps and models for the EM analysis have been deposited to the Electron Microscopy Databank (EMDB) (http://www.emdatabank.org/) and Protein Data Bank (PDB) (http://www.rcsb.org/), respectively. EMDB IDs: 44655-65, 44667-70, 44679-80, 44682-83. PDB IDs: 9BLK and 9BTO. Final statistics and PDB/EMDB deposition

codes are stated in Table S1 and all ns-EM EMDB accession numbers are listed in Table S2. This paper does not report original code. Any additional information required to reanalyze the data reported in this paper, including raw micrographs and/or particle stacks, is available from the lead contact upon request.

Research involving human participants, their data, or biological material

Policy information about studies with human participants or human data. See also policy information about sex, gender (identity/presentation), and sexual orientation and race, ethnicity and racism.

Reporting on sex and gender	n/a
Reporting on race, ethnicity, or other socially relevant groupings	n/a
Population characteristics	n/a
Recruitment	n/a
Ethics oversight	De-identified plasma/sera samples were obtained from UCSD and Yale University as described in the manuscript. Samples were shared for secondary research purposes as permitted by the Informed Consents used upon study enrollment.

Note that full information on the approval of the study protocol must also be provided in the manuscript.

Field-specific reporting

Please select the one below that is the best fit for your research. If you are not sure, read the appropriate sections before making your selection.

☒ Life sciences ☐ Behavioural & social sciences ☐ Ecological, evolutionary & environmental sciences

For a reference copy of the document with all sections, see nature.com/documents/nr-reporting-summary-flat.pdf

Life sciences study design

All studies must disclose on these points even when the disclosure is negative.

Sample size	Sizes of sample groups were minimized for sample availability concerns but still provide statistical significance. For ELISA and HAI assays, the experiments were performed in triplicates (n=3) which is a field-accepted standard for these types of experiments.
Data exclusions	No data was excluded for analysis.
Replication	ELISA, HAI and glycoprotein immobilization experiments were repeated in triplicates (n=3). All EMPEM experiments were performed once (n=1). No Data was excluded.
Randomization	Patient samples were selected in a randomized manner based on serum/plasma availability. Randomization is not applicable to the experiments performed in this study since we were not directly comparing responses between individuals or animals. Rather, we compared two methods for evaluation and no statistical conclusions were drawn.
Blinding	We were blinded to identifying information for all donor samples and therefore this information did not inform us in any way in choosing donor samples.

Reporting for specific materials, systems and methods

We require information from authors about some types of materials, experimental systems and methods used in many studies. Here, indicate whether each material, system or method listed is relevant to your study. If you are not sure if a list item applies to your research, read the appropriate section before selecting a response.

Materials & experimental systems

n/a	Involved in the study	
☐	☒	Antibodies
☐	☒	Eukaryotic cell lines
☒	☐	Palaeontology and archaeology
☐	☒	Animals and other organisms
☒	☐	Clinical data
☒	☐	Dual use research of concern
☒	☐	Plants

Methods

n/a	Involved in the study	
☒	☐	ChIP-seq
☒	☐	Flow cytometry
☒	☐	MRI-based neuroimaging

Antibodies

Antibodies used	Antibodies were isolated and purified from human and animal sera samples. mAbs CC6.30.2 and TXG-0078 were recombinantly expressed and purified as previously described and properly referenced. AP-conjugated AffiniPure goat anti-human IgG was used for ELISA experiments and was purchased from Jackson Immunoresearch (Cat # 109-055-097, Lot # 141947).
Validation	No novel antibodies were discovered or validated.

Eukaryotic cell lines

Policy information about cell lines and Sex and Gender in Research

Cell line source(s)	FreeStyle293F (ThermoFisher Sci, Cat # A14528)
Authentication	No authentication.
Mycoplasma contamination	Mycoplasma is tested on a monthly basis. All cell lines used are confirmed negative.
Commonly misidentified lines (See ICLAC register)	No commonly misidentified cell lines were used in the study.

Animals and other research organisms

Policy information about studies involving animals; ARRIVE guidelines recommended for reporting animal research, and Sex and Gender in Research

Laboratory animals	Healthy adult male or female C57BL/6J (CD45.2+/+) mice heterozygous for the BG18gH KI, generated as previously reported (39), and 8- to 12- week-old male B6.SJL-Ptprca Pepcb/BoyJ mice (CD45.1+/+) purchased from Jackson Laboratory were used in this study. Mice were housed at the animal facility, with free access to food and water, controlled temperature, and a 12:12 hours light-dark cycle. Mice were not involved in previous procedures and were drug and test naïve.
Wild animals	No wild animals were used in the study.
Reporting on sex	Male and Female.
Field-collected samples	No field collected samples were used in the study.
Ethics oversight	Institutional Animal Care and Use Committee (IACUC) of Harvard University and Massachusetts General Hospital (MGH), an Association for Assessment and Accreditation of Laboratory Animal Care International (AAALAC)–accredited facility, under Animal Study Protocols 2016N000286 and 2016N000022.

Note that full information on the approval of the study protocol must also be provided in the manuscript.

Plants

Seed stocks	n/a
Novel plant genotypes	n/a
Authentication	n/a
